# Nebulized hypertonic saline 3% for 1 versus 3 days in hospitalized bronchiolitis: a blinded non-inferiority randomized controlled trial

**DOI:** 10.1186/s12887-019-1804-0

**Published:** 2019-11-08

**Authors:** Gaëlle Beal, Catherine Barbier, Sophie Thoret, Amandine Rubio, Mathilde Bonnet, Roseline Mazet, Anne Ego, Isabelle Pin

**Affiliations:** 1 Department of Pediatrics, Centre Hospitalier Métropole Savoie, FR-73000 Chambéry, France; 2 Department of Pediatrics, Centre Hospitalier Universitaire Grenoble Alpes, FR-38000 Grenoble, France; 3 CIC U1406, INSERM, FR-38000 Grenoble, France; 4 Université Grenoble Alpes, FR-38000 Grenoble, France; 5 Department of Pharmacy, CHU Grenoble Alpes, FR-38000 Grenoble, France; 6 Public Health Department, Université Grenoble Alpes, CNRS, CHU Grenoble Alpes, F-38000 Grenoble, France; 7 Grenoble INP Institute of Engineering, Université Grenoble Alpes, TIMC-IMAG, FR-38000 Grenoble, France

**Keywords:** Bronchiolitis, Hypertonic saline, Children

## Abstract

**Background:**

The use and optimal duration of treatment with nebulized hypertonic saline (HS) in infants hospitalized for acute bronchiolitis is unclear. The objective was to compare the efficacy of 1 versus 3 days of nebulized 3% HS at 72 h of treatment. We conducted a blinded non-inferiority randomized controlled trial including infants aged less than 12 months old, hospitalized for a moderate bronchiolitis.

**Methods:**

Nebulisations of 3% HS for 1 day were followed by either the continuation of 3% HS (HS3d group) or switched to 0.9% normal isotonic saline (HS1d group) for 2 days Randomization was performed according to a predefined list with a 1:1 ratio, obtained with a random generator number with blocks.. Main outcome was mean Wang clinical severity score (CSS) after 72 h of treatment.

**Results:**

One hundred sixteen infants (HS1d *n* = 59 and HS3d *n* = 57), were included over two epidemic seasons from 2014 to 2016, but recruitement did not reach the planned sample size. The difference for the Wang CSS score in the HS3d vs HS1d group was 0.71 [IC 90% 0.1; 1.3], above the precluded value of 0.4 set in the protocol defining the non-inferiority of shorter treatment duration. Clinical remission was more rapidly obtained in the HS3d than in HS1d (2.3 ± 1.6 vs 2.9 ± 1.4 days, *p* = 0.04), with a non-significant tendency for less need of nutritional support and supplemental oxygen in HS3d group. Clinical worsening and treatment intolerance were similar in the 2 groups.

**Conclusions:**

Despite being underpowered, results seem not to be in favour of reducing the duration of nebulised HS treatment from 3 to 1 day in acute moderate bronchiolitis.

**Trial registration:**

Clinical trials NCT 02538458, October 2014.

## Background

Bronchiolitis is one of the most frequent diseases in children under 2 years of age, with about 30% of infants being affected each year, and is a major cause for admission to pediatric emergency departments (ED). Hospitalization rate is about 18 infants per 1000 per year in France [1]. Very few treatments have shown to be effective in the management of this disorder. Treatment is based on supportive care, since previous studies have not shown clearbenefit from corticosteroids, nebulized epinephrine or bronchodilators [2].

In the past 10 years, nebulized hypertonic saline (HS) has emerged as a potentially effective treatment. Nebulized HS is supposed to improve mucociliary clearance, inducing an osmotic flow of water into the mucus layer and rehydrating the airway surface liquid [3]. Among the severity scores used to monitor respiratory outcomes, the Wang Clinical Severity Score (CSS) is widely used and is based on respiratory rate, wheezing, signs of retraction and general condition [4].

Some recent meta-analyses of several clinical studies including the latest Cochrane publication have shown that nebulized HS may reduce length of stay and improve clinical severity scores [5–7]. Despite these data, other meta-analyses suggest that heterogeneity of the studies may not reveal a significant effect on length of stay [8]. However, the ideal duration and timing of administration have yet to be determined, and protocols vary greatly between studies. The most recent guidelines from the American Academy of Pediatrics recommend that nebulized HS should not be administered to infants with a diagnosis of bronchiolitis in the Emergency Department (ED) but only for patients hospitalized for bronchiolitis (Grade B recommendation) [9]. Two very recent studies also showed that there was no benefit from using 3% HS in the pediatric ED [10, 11]. A meta-analysis by Chen et al. [12] suggests that the benefit on the clinical severity score exists as early as on the first day of treatment and that it is still significant on the third day.

Considering that the optimal duration of nebulized HS in infants hospitalized for acute bronchiolitis is still debated, we conducted a blinded non-inferiority randomized controlled trial aimed to compare nebulized 3% HS for 1 versus 3 days on severity score and clinical remission at 72 h.

## Methods

### Trial design

We conducted a non-inferiority, randomized, double-blind, controlled trial in infants hospitalized for viral bronchiolitis in the pediatric department of the Grenoble Alpes University Hospital. Children were screened from the pediatric ED during the epidemic seasons of bronchiolitis in 2014 and 2015 and included at the end of the first 24 h following admission. Follow-up was maintained until discharge from hospital. Background, demographic information and medical data were collected through standardized case record forms. Study endpoints were based on data collected at clinical examinations performed at 24, 48 and 72 h of treatment and at the time of discharge. There was no change in routine bronchiolitis monitoring or supplementary exams. Viral research was not routinely performed.

### Participants

The eligible population was infants aged less than 12 months, admitted to the pediatric ED, for a moderate bronchiolitis (Wang CSS at admission from 4 to 9), requiring hospitalization. Bronchiolitis was defined at admission as the first or second episode ever of cough with increased respiratory effort and wheezing or crackles, following an upper respiratory tract infection. Exclusion criteria were based on medical history (underlying chronic cardiopulmonary or neurological diseases, premature birth below 34 weeks of gestational age, asthma (3rd wheezing episode or more)), severe bronchiolitis (either Wang CSS > 9 or baseline oxygen saturation < 85%), indication for transfer to the Pediatric Intensive Care Unit in the first 24 h, treatment continuation with inhaled corticosteroids and/or bronchodilators. Information was provided to parents at admission after first emergency room examination, and the recruitment was completed in the first 24 h of hospitalization, allowing a time for reflection for parents.

### Randomization and interventions

During the first 24 h, nebulizations were performed with 4 ml of 3% HS administered every 8 h. Eligible patients were then randomized to either the continuation of 3% HS (HS3d group) or the switch to 0.9% normal isotonic saline (NS, HS1d group) administered every 8 h for the next 2 days. Randomization was performed by the pharmaceutical department of the hospital, which delivered sequentially numbered required containers according to a predefined list with a 1:1 ratio, obtained with a random generator number with blocks.

The HS and NS 4 ml solutions were indistinguishable by aspect and smell. The composition of study solutions and random assignment of patients to intervention group (HS1d) or control group (HS3d) was blinded to all study subjects, parents/guardians, medical care providers, and investigators. The solution was administered via a pediatric Eco Aerosol Cirrus 2 nebulizer (Intersurgical®) and a face mask with an oxygen flow of 6–8 l/min. The nebulization was continued until the nebulization chamber was empty. Physical examination was carried out twice daily by the physician in charge of the child who was blinded to the exact nature of the treatment. Oral corticosteroids were authorized if indicated as an adjuvant therapy for ventilation disorders present on chest X-ray. If infants met early discharge criteria, an additional consultation was scheduled at 72 h with one of the study investigator to evaluate the primary outcome.

### Outcomes

The primary outcome was the Wang CSS after 72 h of treatment, including the first 24 h of open 3% HS treatment, and the 48 h of blinded study treatment with either 3% HS or NS nebulizations. Secondary outcomes were 1) daily Wang CSS evolution from time of admission to 72 h of treatment and 2) clinical remission, defined by all the following items: oxygen saturation > 92% during wakefulness and > 90% during sleep, spontaneous feeding > 2/3 of usual portion during the last 2 meals, respiratory rate < 60/min and CSS < 4. The delay to clinical remission was used rather than the length of stay, because less sensitive to socio-economic or logistic factors. Other secondary endpoints were requirement for oxygen supplementation (if oxygen saturation was < 92% during wakefulness or < 90% during sleep for at least 5 min) and for enteral nutrition by nasogastric tube at any time (if food intake was less than 50% of the expected portions for weight on two consecutive meals).

Adverse events were recorded: clinical worsening was defined as a Wang CSS > 9 at any time, or the necessity for a transfer to the Pediatric Intensive Care Unit (PICU), or the need for ventilatory support (Wang CSS > 9, pH < 7.35 or venous PCO2 > 50 mmHg) or the requirement for additional treatment such as inhaled bronchodilators. Extended length of stay was defined as a length of stay greater than 6 days. Treatment intolerance was defined as excessive cough or desaturation during nebulization, or any clinically significant event related to the nebulization. The clinical examination and calculation of Wang’s score was done by the doctor in charge of the child in the unit. All the investigators were blinded to treatment.

### Sample size

Given a mean Wang CSS after 3 days of admission in the control group of 3–4 in the literature [6, 12] and a standard deviation of 1, non-inferiority was defined prior to the study by a less favorable score in the HS1d group within a relative increase of ≤10%, ie + 0.4 point of the score. In our experience, this difference corresponds to a loss of effectiveness that seems clinically negligible for the patient. To achieve 80% power with a one-sided hypothesis of 5% (corresponding to a 90% confidence interval), 78 patients were needed in each group.

### Statistics

Qualitative variables were noted as numbers and percentages, and quantitative variables as means and standard deviation. Baseline patient demographic and clinical characteristics between intervention and control groups were compared with Student test for quantitative variables, and χ^2^ or Fisher exact test for qualitative variables. The Wang CSS difference described by mean and 90% confidence interval was evaluated in a per-protocol analysis while secondary outcomes were analyzed in relation to intention-to-treat. The evolution of the average CSS score between the 2 arms was compared using an analysis of variance for repeated measures. *P*-values less than 0.05 were considered statistically significant and analyses were performed with Stata software version 13 (Stata Corp 4905 Lakeway Drive College Station, TX 77845 USA).

## Results

**Fig. 1:**
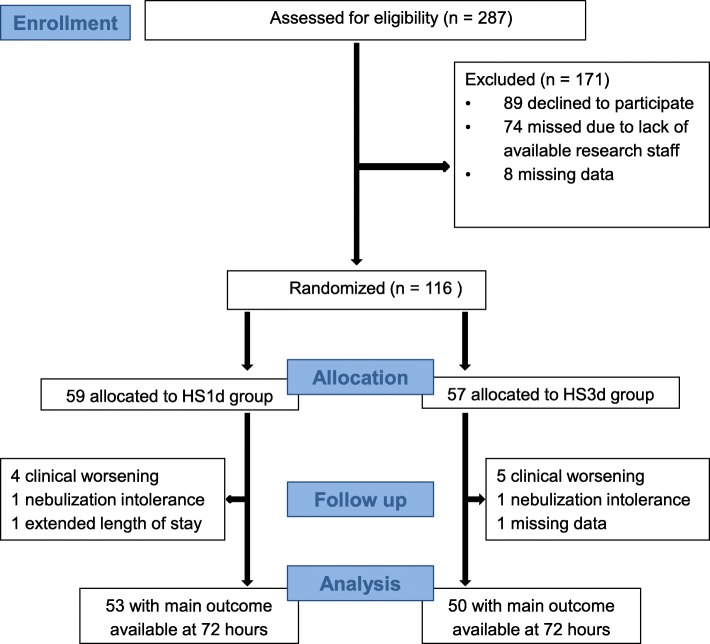
CONSORT flow diagram

During the recruitment period, 287 eligible patients were admitted for a moderate bronchiolitis (Fig. 1). Among them, 89 declined to participate, 74 were not approached due to unavailability of research staffand information at admission was missing from medical records for the last 8 patients. The remaining sample of 116 patients was randomized: 59 to the HS1d group, and 57 to the HS3d group. Six patients in the HS1d group and 7 patients in the HS3d group presented adverse outcomes leading to interruption of nebulizations according to the protocol, leading to 103 patients, 53 and 50 respectively in the HS1d and HS3d groups with available main outcomes at 72 h. Mean age was 4.2 +/− 2.4 months in the overall sample. Patients in both groups had moderate bronchiolitis at randomization with a mean Wang CSS of 5.7 ± 1.8, which was inferior to the mean Wang CSS at admission (6.6 ± 1.1). Twelve percent of infants had already had a previous bronchiolitis. There was no statistical difference between intervention and control groups as regard gender, gestational age and birthweight, risk factors for bronchiolitis, and severity of respiratory symptoms at admission (Table 1).

**Table 1:** Baseline characteristics by treatment allocation

Characteristics	*n*	Total	HS1d *n* = 59	HS3d *n* = 57	*p*-value
Gender (male n, %)	116	76 (65.5)	34 (57.3)	42 (73.7)	0.069
Age in month (mean ± SD)	116	4.2 ± 2.4	4.1 ± 2.5	4.4 ± 2.4	0.427
*Gestational age in weeks* (mean ± SD)	107	38.8 ± 4.0	38.4 ± 5.5	39.1 ± 1.4	0.378
Birthweight in g (mean ± SD)	116	3340 ± 475	3299 ± 474	3382 ± 477	0.357
Weight in g (mean ± SD)	110	6452 ± 1509	6290 ± 1456	6619 ± 1558	0.24
Environmental smoke exposure (n, %)	116				
Current		32 (27.6)	17 (28.8)	(26.3)	0.763
During pregnancy		18 (15.5)	9 (15.2)	9 (15.8)	0.916
Personal history of atopy (n, %)	116	13 (11.2)	4 (6.8)	9 (15.7)	0.124
Familial history of atopy (n, %)	116	70 (60.3)	33 (55.9)	37 (64.9)	0.323
Previous episode of bronchiolitis (n, %)	116	14 (12.1)	6 (10.2)	8 (14)	0.523
CSS at randomization (mean ± SD)	116	5.7 ± 1.8	5.8 ± 1.7	5.5 ± 1.8	0.403
Associated treatments at admission (n, %)	116				
Inhaled beta2agonists		14 (12.1)	9 (15.2)	5 (8.8)	0.284
Antibiotics		33 (28.4)	17 (28.8)	16 (28.1)	0.929
Paracetamol		50 (43.1)	37 (45.8)	23 (40.3)	0.556

**Fig. 2:**
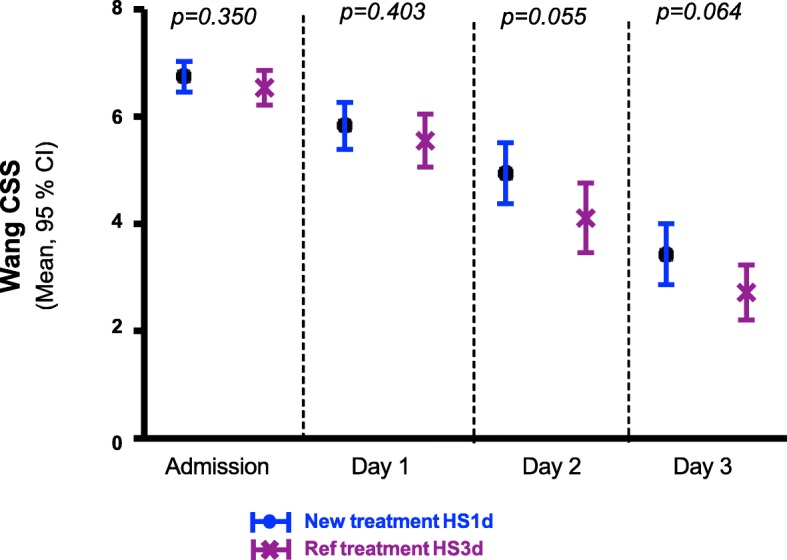
Wang CSS evolution by group

Table 2 summarizes the primary and secondary endpoints. The mean difference for the Wang CSS at 72 h in the HS3d vs HS1d arm was 0.7 [IC 90% 0.1; 1.3], with respective values of 2.7 ± 1.8 vs 3.4 ± 2.1. The higher boundary being greater than the specified non-inferiority margin set as 0.4, the non-inferiority hypothesis was statistically rejected. The groups were therefore compared according to a superiority hypothesis. Figure 2 represents daily average Wang CSS evolution for the 2 groups. Wang CSS decreased in both groups. There was a tendency for difference between the two groups in favor of the HS3d group on the second (*p* = 0.055) and third day (*p* = 0.064) of treatment. The average CSS score between the two arms, compared by using an analysis of variance for repeated measures, was not statistically different (*p* = 0.262).

**Table 2:** Primary and secondary outcomes

	HS1d	HS3d	Difference (90% CI)	*p*-value
Primary outcome	*n* = 53	*n* = 50		
Wang CSS at 72 h of treatment (mean ± SD)	3,4 ± 2,0	2.7 ± 1.7	0.7 (0.1–1.3)	0.064
Secondary outcomes	*n* = 59	*n* = 57		
Wang CSS at 48 h of treatment (mean ± SD)	4.9 ± 2.1	4.1 ± 2.4		0.055
Clinical remission in days (mean ± SD)	2.9 ± 1.4	2.2 ± 1.6		0.043
Enteral nutrition (n, %)	29 (49.1)	21 (36.8)		0.181
Oxygen supplementation (n, %)	30 (50.8)	21 (36.8)		0.129

Time to clinical remission was statistically significantly lower in the HS3d group compared to the HS1d group (2.9 ± 1.4 vs 2.2 ± 1.6 days, *p* = 0.04). No statistically significant differences were observed for the need for oxygen supplementation or enteral nutrition (Table 2).

There was no difference in the rates of clinical worsening, intolerance of nebulization and extended length of stay between intervention and control groups (Table 3). Only one child was transferred to PICU for clinical worsening just after randomization, before receiving the study treatment. Most cases of nebulization intolerance were by worsening of cough during nebulization. There was only one case of treatment intolerance in the HS3d group, with desaturation during nebulization. One child presented a latex contact dermatitis due to the nebulization mask. Extended lengths of stay were all due to oral feeding difficulties after enteral nutrition with nasogastric tubes.

**Table 3:** Tolerance study

	Total *n* = 116	HS1d *n* = 59	HS3d *n* = 57	*p*-value
Clinical worsening^a^ (n, %)	11 (9.5)	5 (8.4)	6 (10.5)	0.706
Length of stay > 6 days (n, %)	6 (5.2)	3 (5)	3 (5.2)	1.000
Treatment intolerance^b^ (n, %)	5 (4.3)	3 (5)	1 (1.7)	0.745

^a^Wang CSS > 9 at any time, necessity for a transfer to the PICU, need for ventilatory support, requirement for additional treatment such as inhaled bronchodilators

^b^Excessive cough or desaturation during nebulization, or any clinically significant event related to the nebulization

## Discussion

### Summary of findings

This prospective, non-inferiority, randomized double-blind controlled trial comparing 1 vs 3 days of 3% HS nebulized treatment in hospitalized children < 12 months old with moderate bronchiolitis is not in favor of shorter duration of treatment, with a tendency for worse Wang scores at 2 and 3 days and significantly longer duration of clinical remission if the group treated for 1 day.

### Limitations

The authors acknowledge some limitations of this study. The study was designed and performed at a period (2014–2015) when HS treatment for bronchiolitis was less controversial. Due to recruitment difficulties (lack of funding did not allow a third recruitment season), we did not achieve the expected number of inclusions.

Despite this lack of statistical power, our results are in favor of continuing 3% HS nebulization in children hospitalized for moderate bronchiolitis for more than 24 h. Lack of power is likely to explain the non-statistically significant tendency for the comparison of the Wang CSS at 48 and 72 h.

We acknowledge that only a minority of eligible children could be included in the study. The reasons for this, due primarily to the short delay and some reluctance of parents for obtaining consent, are unlikely to induce a recruitment bias. Despite the single-center design of our trial, likely to limit the generalizability of our findings, comparable treatment strategies are currently applied in many pediatric departments and there are no arguments to support the specific nature of our recruitment, organization, or medical care.

We voluntarily did not include children whose clinical evolution allowed discharge within the first 24 h, assuming that this population corresponded to mild bronchiolitis or simple rhinitis with a diagnostic error. Infants with severe acute bronchitis who required direct PICU admission and patients with milder forms of acute bronchiolitis were excluded. We cannot exclude that nebulized HS could have be useful in these populations.

Nebulized NS is not a neutral product and could have an  influence on respiratory scores [13, 14]. Our choice to use it was based on the necessity to maintain a double blind design in our study. Most of the double blind trials studying the effect of HS used NS as comparator [10, 15–20]. Only one trial was carried out with absence of NS as comparator [21], and another with the absence of blinding [20]. We cannot exclude that neither intervention is beneficial because a placebo arm is missing in the current trial.

### Comparison with the literature

When initial trials for nebulized HS delivery appeared in the literature, there was hope that it would be used to speed recovery time and shorten the length of stay (LOS) [5]. Since then, there has been extensive research in the literature regarding HS nebulization in acute bronchiolitis. Conclusions regarding the effectiveness of HS nebulizations are still debated [22, 23]. In the past years, several trials about efficacy of 3% HS nebulization in hospitalized patients were published [15, 16, 21, 24]. Seven different meta-analyses were published including the last Cochrane review [7], still yielding contradictory results [6, 8, 13, 14, 24, 25]. A very recent study by Harrison and al. used a new analytic technique called trial sequential analysis (TSA) [22]. Unfortunately, despite a large number of studies, there are still not enough data to draw firm conclusions regarding the effectiveness (or lack of effectiveness) of HS. However, as regards the “length of stay” outcome, the cumulative *z* score is close to the « monitoring boundary », which is the point in TSA at which a significant difference can be concluded.

The use of HS in acute bronchiolitis is therefore still controversial. Disparity in the definition of bronchiolitis, inclusion criteria, concomitant drugs administration, and differences in outcome measures are likely to account for the discrepancy of these results. In 2014, Barben et al. emphasized the importance of using the same definition for bronchiolitis [26]. Our study respected the suggested inclusion criteria, namely infants aged < 12 months, with typical clinical presentation. It was possible to include infants with at most one previous episode of wheeze, assuming that the limit of a single previous episode decreased the possibility of enrolling patients with asthma. These patients represented only 10% of our population, and there was no difference between the two groups. The use of varying bronchodilators was not controlled in the original studies, and the use of different clinical scoring systems led to varying inclusion criteria in terms of disease severity and varying outcome assessments. Our study adds new data to the discussion, as it is the first to study the optimal duration of 3% HS nebulization in a controlled study. It shows that 3 days of HS in moderate bronchiolitis seems to be more effective than 1 day and the benefit seems to be present already at 2 days of treatment. Most trials showing a reduction of length of stay by HS nebulization were carried out in hospitalized patients, with administration of HS from 3 days to clinical remission of the child [17, 19]. We choose to limit the duration of the treatment for 3 days maximum, assuming that the natural course of bronchiolitis beyond this period tends towards clinical improvement. Considering treatment secondary effects, there was only one case of treatment intolerance in the HS3d group, in favor of a good tolerance of 3% HS nebulization, as previously described [27].

## Conclusions

Despite being underpowered, this study led to consistent results that seem to favour 3% HS treatment over 3 days rather than 1 day in acute moderate hospitalized bronchiolitis. Tolerance of 3% HS nebulizations is good. However, other studies with larger standardized samples and multicenter designs would be necessary to definitively conclude whether HS nebulizations have clinically significant effects or not.

## Data Availability

The datasets used and/or analyzed during the current study are available from the corresponding author on reasonable request.

## Abbreviations

CSS: Clinical Severity Score
ED: Emergency Department
HS: Hypertonic Saline
LOS: Length Of Stay
PICU: Pediatric Intensive Care Unit
TSA: Trial Sequential Analysis
